# Transcription modulation by CDK9 regulates inflammatory genes and RIPK3-MLKL-mediated necroptosis in periodontitis progression

**DOI:** 10.1038/s41598-019-53910-y

**Published:** 2019-11-22

**Authors:** Jiao Li, Jiahong Shi, Yue Pan, Yunhe Zhao, Fuhua Yan, Houxuan Li, Lang Lei

**Affiliations:** 1 Department of Orthodontics, Nanjing Stomatological Hospital, Medical School of Nanjing University, Nanjing, China; 2 Central Laboratory of Stomatology, Nanjing Stomatological Hospital, Medical School of Nanjing University, Nanjing, China; 3 Department of Periodontics, Nanjing Stomatological Hospital, Medical School of Nanjing University, Nanjing, China

**Keywords:** Necroptosis, Periodontitis, Chronic inflammation

## Abstract

Cyclin-dependent kinase 9 (CDK9), one crucial molecule in promoting the transition from transcription pausing to elongation, is a critical modulator of cell survival and death. However, the pathological function of CDK9 in bacterial inflammatory diseases has never been explored. CDK9 inhibition or knock-down attenuated *Porphyromonas gingivalis-*triggered inflammatory gene expression. Gene-expression microarray analysis of monocytes revealed that knock-down of CDK9 not only affected inflammatory responses, but also impacted cell death network, especially the receptor-interacting protein kinase 3 (RIPK3)-mixed lineage kinase domain-like (MLKL)-mediated necroptosis after *P. gingivalis* infection. Inhibition of CDK9 significantly decreased necroptosis with downregulation of both MLKL and phosphorylated MLKL. By regulating caspase-8 and cellular FLICE inhibitory protein (cFLIP), key molecules in regulating cell survival and death, CDK9 affected not only the classic RIPK1-RIPK3-mediated necroptosis, but also the alternate TIR-domain-containing adapter-inducing interferon-β-RIPK3-mediated necroptosis. CDK9 inhibition dampened pro-inflammatory gene production in the acute infection process in the subcutaneous chamber model *in vivo*. Moreover, CDK9 inhibition contributed to the decreased periodontal bone loss and inflammatory response induced by *P. gingivalis* in the periodontal micro-environment. In conclusion, by modulating the RIPK3-MLKL-mediated necroptosis, CDK9 inhibition provided a novel mechanism to impact the progress of bacterial infection in the periodontal milieu.

## Introduction

Periodontitis is a kind of bacteria-inflicted inflammation in the periodontal tissue. Overdue growth of anaerobic “red complex” bacteria, including *Porphyromonas gingivalis* and *Treponema denticola* has been implicated in both the onset and progression of periodontal diseases^1^. The host sentinel cells, such as monocytes, macrophages and neutrophils, stand at the first line against the invasion of periodontal pathogens. By a vast array of pattern recognition receptors (PRRs), they may bind to pathogen-associated molecular patterns (PAMPs), including lipopolysaccharide (LPS), DNA, RNA, and carbohydrates^2^.

Ligation of PRRs with PAMPs will initiate a cascade of downstream signaling pathway to address the disrupted cellular microenvironment, leading to changes in the PAMP response genes^3^. Such inflammatory response leads to the generation of multiple chemokines to recruit more sentinel cells to sites of inflammation to combat the invasion of bacteria; in addition, production of pro-inflammatory mediators, such as tumor necrosis factor α (TNF-α) and transforming growth factor-β (TGF-β) may facilitate survival of host cells to sustain the infection^4^. Moreover, stimuli from invading bacteria may trigger several distinct regulated cell deaths (RCD), such as apoptosis, NETosis, necroptosis and pyroptosis. Generally, the classical apoptosis is not inflammatory as the cell membrane keeps intact, whereas pyroptosis and necroptosis are highly proinflammatory due to the rupture of cell membrane^5^. With its profuse discharge of damage associated molecular patterns (DAMPs), such as interleukin-1α (IL-1α), mitochondria, ribosomes as well as DNAs, necroptotic cell death contributes to amplification of inflammation^6^. In addition, apoptosis or autophagy also participates in the immune response to bacterial infection, contributing to pathogen clearance but not eliciting host inflammation^7^.

Expression of inflammatory mediators and various cell death pathways must be delicately orchestrated to prevent inordinate inflammatory response and tissue destruction. Indeed, several negative regulatory mechanisms that restrain pro-inflammatory cytokine production at multiple levels have been discovered^8^. The complicated nature of transcription process makes the process a proper loci to mount precise and proper inflammatory responses to given environmental cues^9^. The recruitment and binding of RNA Polymerase II with various transcription factors onto transcription start sites (TSS) is an important mechanism for regulating the expression of a myriad of target genes^10^. Shortly after initiation of transcription, such process pauses at the promoter-proximal loci, which is ∼50 nt downstream of TSS. The cyclin-dependent kinase 9 (CDK9) and cyclin T1 facilitate the transition from transcription pausing to elongation via phosphorylation of the C-terminal domain of the RNA polymerase II as well as several negative factors^11^. In addition, CDK9 may coordinate with the Bromodomain-containing protein (Brd) 4 to dynamically enhance the transcription elongation^12,13^.

The balance of cell survival and death in response to bacterial invasion is controlled by key molecules in the innate immune response, including receptor-interacting protein kinase (RIPK) -1, -3, caspase 8 and cFLIP. We postulated that by modulating key molecules in the network of cell survival and death, CDK9 plays a pivotal role in the onset and progress of periodontitis. Our research demonstrated that CDK9 activation regulated the inflammatory gene transcription and RIPK3-mixed lineage kinase domain-like (MLKL)-mediated necroptosis following *P. gingivalis* invasion and further influenced the progress of periodontitis.

## Results

### TOP1, Brd4 and CDK9 expression was increased in chronic periodontitis

**Figure 1:**
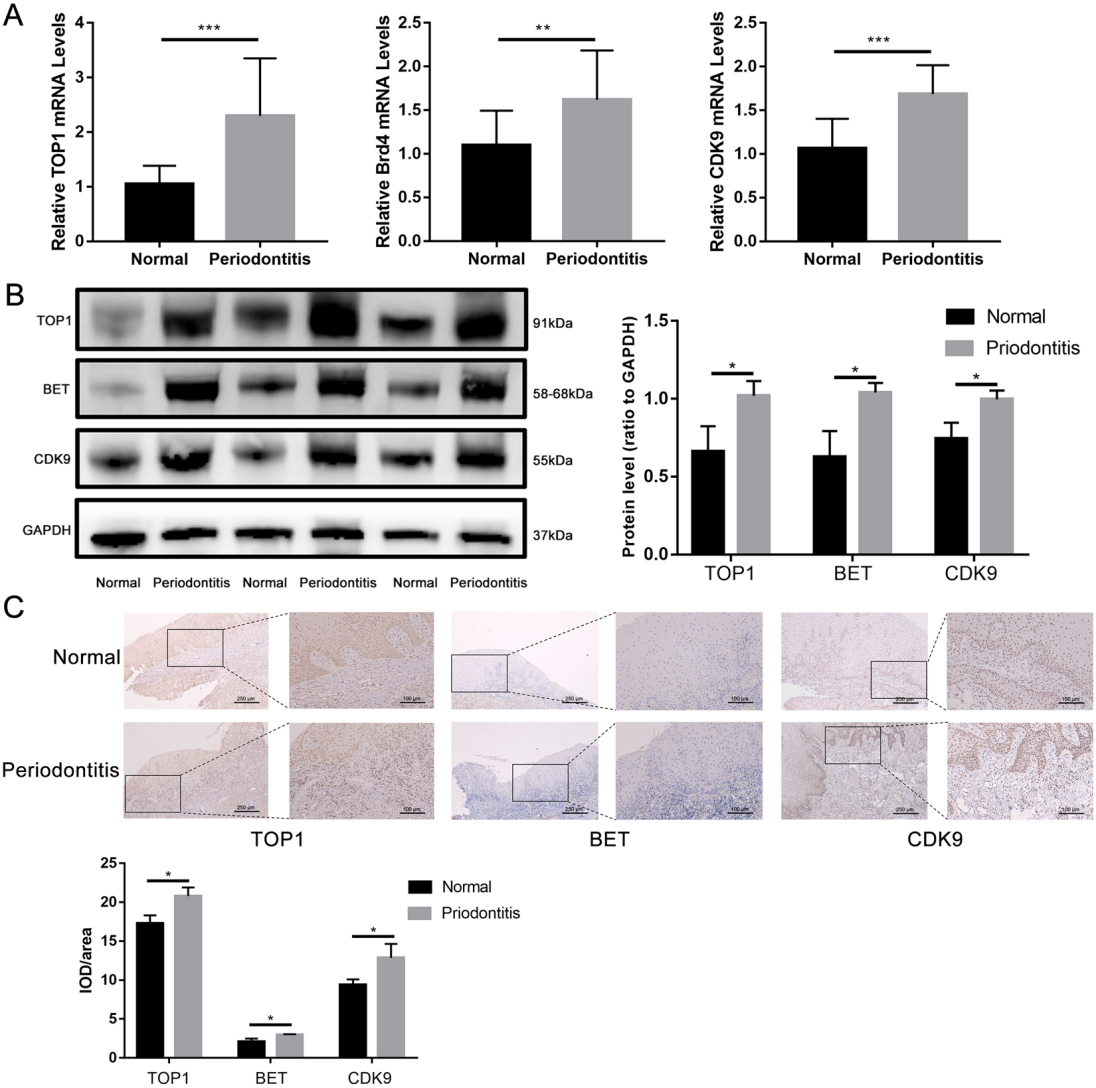
Expression of TOP1, BET and CDK9 was elevated in the inflamed periodontal tissues. Periodontal tissues were collected in the healthy gingiva during exposure of impacted teeth and the inflamed gingiva during extraction of hopeless teeth. (**A**) The transcriptional level of TOP1, Brd4 and CDK9 in the periodontal tissues was quantified by qPCR; (**B**,**C**) protein expression of TOP1, BET and CDK9 was measured by Western blot and immunohistochemistry. The images were collected from different gels with the same amount of samples. (**p < 0.01; **p < 0.001).

Brd4 and CDK9-mediated gene transcription has been implicated in inflammatory diseases such as radiation-induced lung fibrosis in mice^14^ and inflammatory process in the placentas of patients with preeclampsia^15^. We postulated that activation of TOP1, Brd4 and CDK9-mediated gene transcription may accompany the periodontal destruction in the periodontium. We observed nearly 1-fold increase in the TOP1 mRNA transcription, ~50% increase in the Brd4 and CDK9 transcription in the inflamed gingiva when compared to the healthy control (Fig. 1A). Minor protein expression of TOP1, Brd4 and CDK9 can be found in the healthy gingiva, while significant higher protein expression can be detected in the diseased periodontal tissues by Western blot, indicating robust gene transcriptions of inflammatory genes in the periodontal biopsies (Fig. 1B). To further investigate their role in the periodontal inflammation, we also conducted immunohistochemical staining, finding that increased expression of the three proteins in both the gingival epithelium and subepithelial connective tissue in the inflamed gingiva, further indicating that TOP1, Brd4 and CDK9 participated in the progression of periodontal diseases (Fig. 1C).

### Inhibition of CDK9 contributed to reduced cytokine production in response to bacterial infection

**Figure 2:**
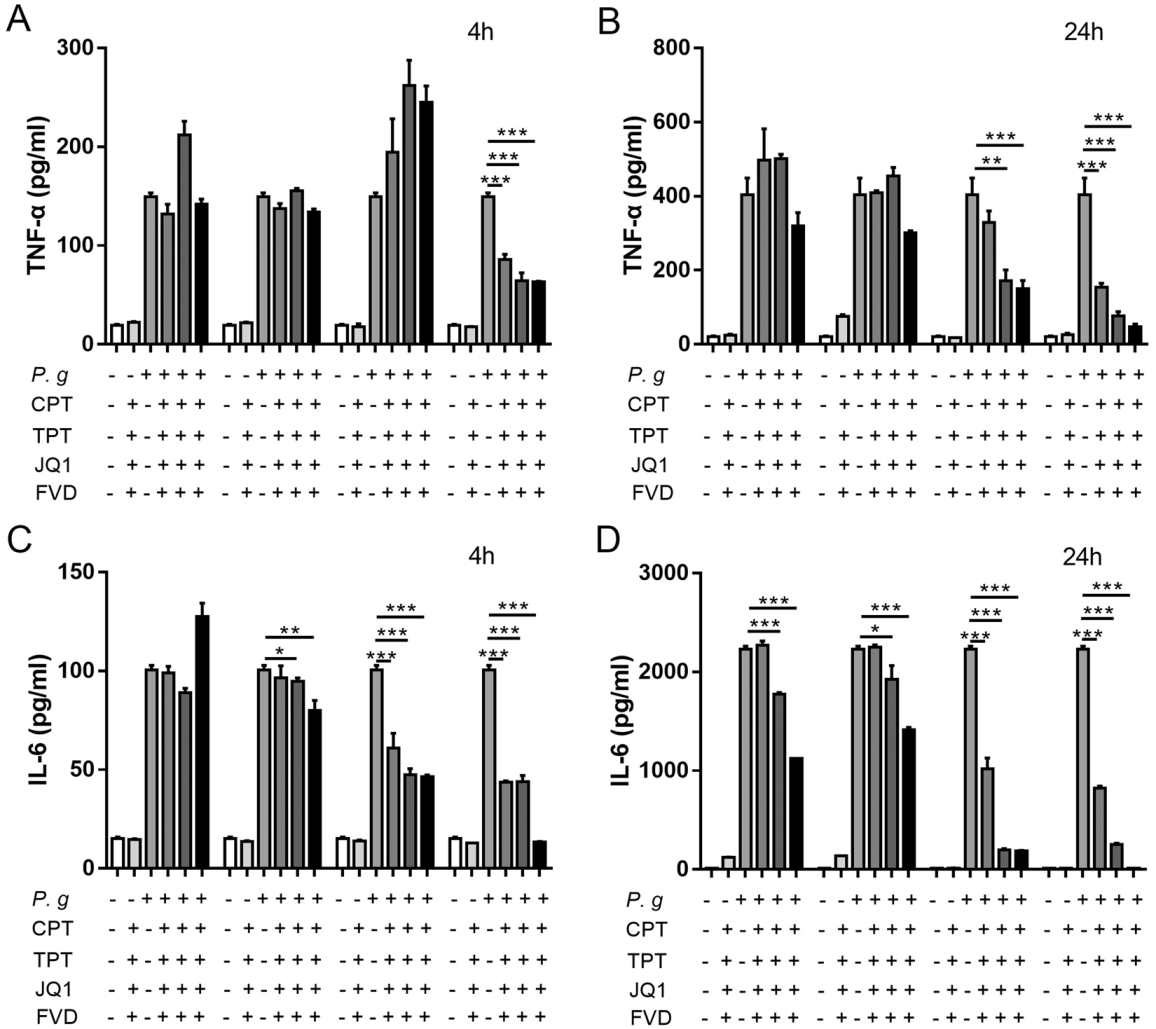
Transcription modulation affected expression of inflammatory cytokines. THP-1 cells were pretreated with CPT, TPT, JQ1 and FVD for 2 hours and stimulated with *P. gingivalis*. Production of (**A**,**B**) TNF-α and (**C**,**D**) IL-6 in culture supernatants at 4 and 24 hours after infection was detected by ELISA. (*p < 0.05; **p < 0.01; ***p < 0.001).

Defense cells in the innate immune system may tackle the outburst of pathogens via a coordinated expression of early response and late response genes^16^. To explore the effect of TOP1, Brd4 and CDK9 activation in inflammation, we screened the effects of inhibition of TOP1, Brd4 and CDK9 in the pro-inflammatory responses to *P. gingivalis*. THP-1 monocytes were pretreated with Camptothecin (CPT, Selleck) and Topotecan HCl (TPT, Selleck), inhibitors of TOP1, (+)-JQ1 (Selleck), the inhibitor of Brd4, and Flavopiridol (FVD, Selleck), the inhibitor of CDK9. No significant reduction in the proinflammatory cytokine production was observed in the TOP1 inhibition group after *P. gingivalis* stimulation, whereas reduced IL-6 and TNF-α expression were observed after Brd4 or CDK9 inhibition at 4- and 24-hours following *P. gingivalis* infection (Fig. 2). Similar results were found in LPS-treated RAW264.7 macrophages by pretreatment with JQ1 and FVD (see Supplementary Fig. S1). Therefore, targeting Brd4 or CDK9 may be effective in alleviating inflammation in the periodontal tissue.

**Figure 3:**
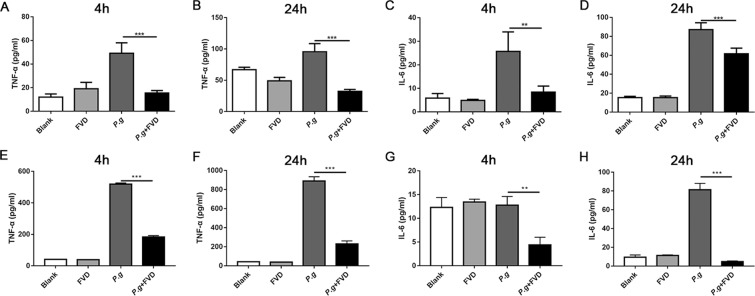
Inhibition of CDK9 attenuated pro-inflammatory genes in monocytes following *P. gingivalis* infection. (**A**–**D**) THP-1 monocytic cells were preincubated with FVD to block CDK9 for 2 hours and then infected with *P. gingivalis*. Production of cytokine were assessed by ELISA at 4 and 24 hours after infection. (**E**–**H**) PBMCs were pretreated with FVD and then infected with *P. gingivalis*. Cytokine production in the culture was detected by ELISA. (**p < 0.01; ***p < 0.001).

The immune system expresses various PRRs, especially TLRs, to detect danger and mount a specific immune response^17^, and inordinate inflammation may lead to tissue destruction in the chronic periodontitis^18^. The innate immune responses to bacterial infection are accompanied by a robust gene transcription^19^; however, the role of CDK9 in bacterial infection has never been explored. Therefore, we explored the role of CDK9 in the periodontal infection. THP-1 monocytic cells were pretreated with FVD to inhibit CDK9, and then stimulated with *P. gingivalis*. TNF-α and IL-6 production was significantly reduced by FVD pretreatment (Fig. 3A–D). Such inhibitory effect was also observed in peripheral blood mononuclear cells (PBMCs) (Fig. 3E–H), indicating that CDK9 activation in monocytes may facilitate the proinflammatory cytokine production. Such results were consistent with the inhibitory effect of CDK9 inhibition in LPS treated chondrocytes^20^.

### CDK9 knock-down dampened inflammatory response and necroptosis in monocytes following *P. gingivalis* invasion

**Figure 4:**
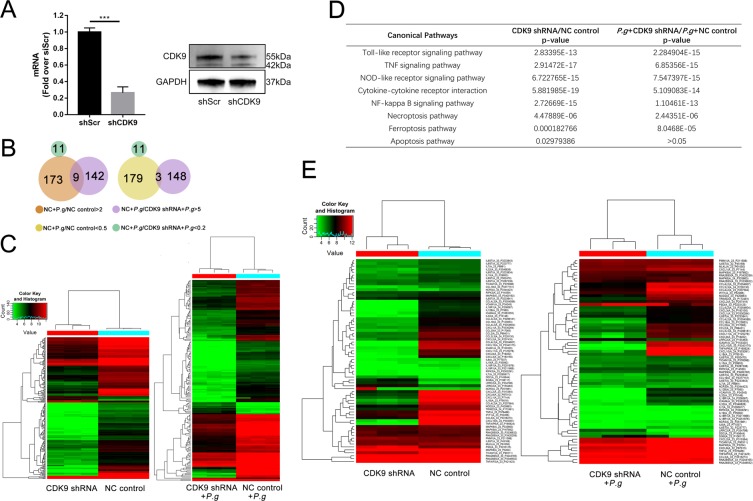
CDK9 knock-down altered gene expression during *P. gingivalis* infection. (**A**) Inhibited RNA and protein level of CDK9 was observed in CDK9 shRNA constructs. The images were collected from different gels with the same amount of samples. (**B**) Venn diagrams were utilized to show logic of differentially-regulated genes(fold change ≥2) between CDK9 knock-down constructs and scramble constructs 2 hours after *P. gingivalis* infection. (**C**) Heat map was utilized to show differentially regulated genes between THP-1 monocytic cells with or without CDK9 knock-down 2 hours after *P. gingivalis* treatment. (**D**) The overall effect of CDK9 knock-down on *P. gingivalis* infection were analyzed by KEGG pathway analysis^77–79^. (**E**) A detailed heat map was used to show differentially-regulated inflammatory genes and cell death genes. (***p < 0.001).

Monocytes are quick responders to stimuli, leading to activation of the classic TLR signaling after exposure to pathogens^21^. Because chemical inhibition may suffer from limited selectivity, we knocked down CDK9 in human THP-1 monocytic cell lines via lentiviral transfection of short hairpin RNA (shRNA). Notably, CDK9 activation is required for transcriptional elongation by Pol II during mRNA transcription^22,23^ and its complete ablation would adversely affect cell viability and animal survival^10^. ~70% inhibition of CDK9 in qPCR and Western Blot (Fig. 4A) did not significantly affect cell viability in our experiment.

To further unveil the contribution of CDK9 during *P. gingivalis* infection, gene chips were performed. Among the genes with altered transcription, we discovered that CDK9 knock-down impacted the expression of a large scale of inflammatory gene after 2 h *P. gingivalis* stimulation, a total of 173 genes were activated, while 179 genes were suppressed by more than twofold with *P. gingivalis*, CDK9 knock-down contributed to the down-regulation of 151 genes by more than fivefold, of which 9 genes were activated by *P. gingivalis*, and contributed to the up-regulation of 11 genes (Fig. 4B).

CDK9 may function as a co-activator and co-repressor of gene transcription^24,25^; consistently, multiple genes were either increased or decreased in CDK9-knock-down THP-1 cells (Fig. 4C). To further characterize the effects of CDK9 activation in the bacterial infection, KEGG pathway analysis was utilized to explore the overall effect of CDK9 knock-down on *P. gingivalis* infection (Fig. 4D), and we discovered that CDK9 knock-down affected multiple inflammatory pathways, including TLR, NLR and TNF signaling pathway. In addition, CDK9 knock-down inhibited gene transcription in necroptosis and ferroptosis pathways whether monocytes were stimulated with bacteria or not. CDK9 knock-down affected apoptosis in monocytes without bacterial infection, while no difference was found upon *P. gingivalis* incubation. The genes related to inflammation and cell death were further displayed in Fig. 4E. Our present data demonstrated that CDK9 regulated defense responses during bacteria invasion by modulating molecules related to inflammation and cell death, especially necroptosis.

### CDK9 modulated both RIPK1-and TRIF-dependent RIPK-MLKL mediated necroptosis

Previously, we have observed that *P. gingivalis* induced cellular necroptosis through RIPK1/RIPK3/MLKL signaling pathway in monocytes, generating profuse discharge of DAMPs^26^. Moreover, pathogens like *Mycobacterium tuberculosis*^27^, *Staphylococcus aureus*^5^ and *Pneumococcal Pneumonia*^28^ manipulates necroptosis for the microbial invasion and disease progression. We then explored the role of necroptosis pathway in CDK9-mediated inflammatory responses against the *P. gingivalis* infection.

**Figure 5:**
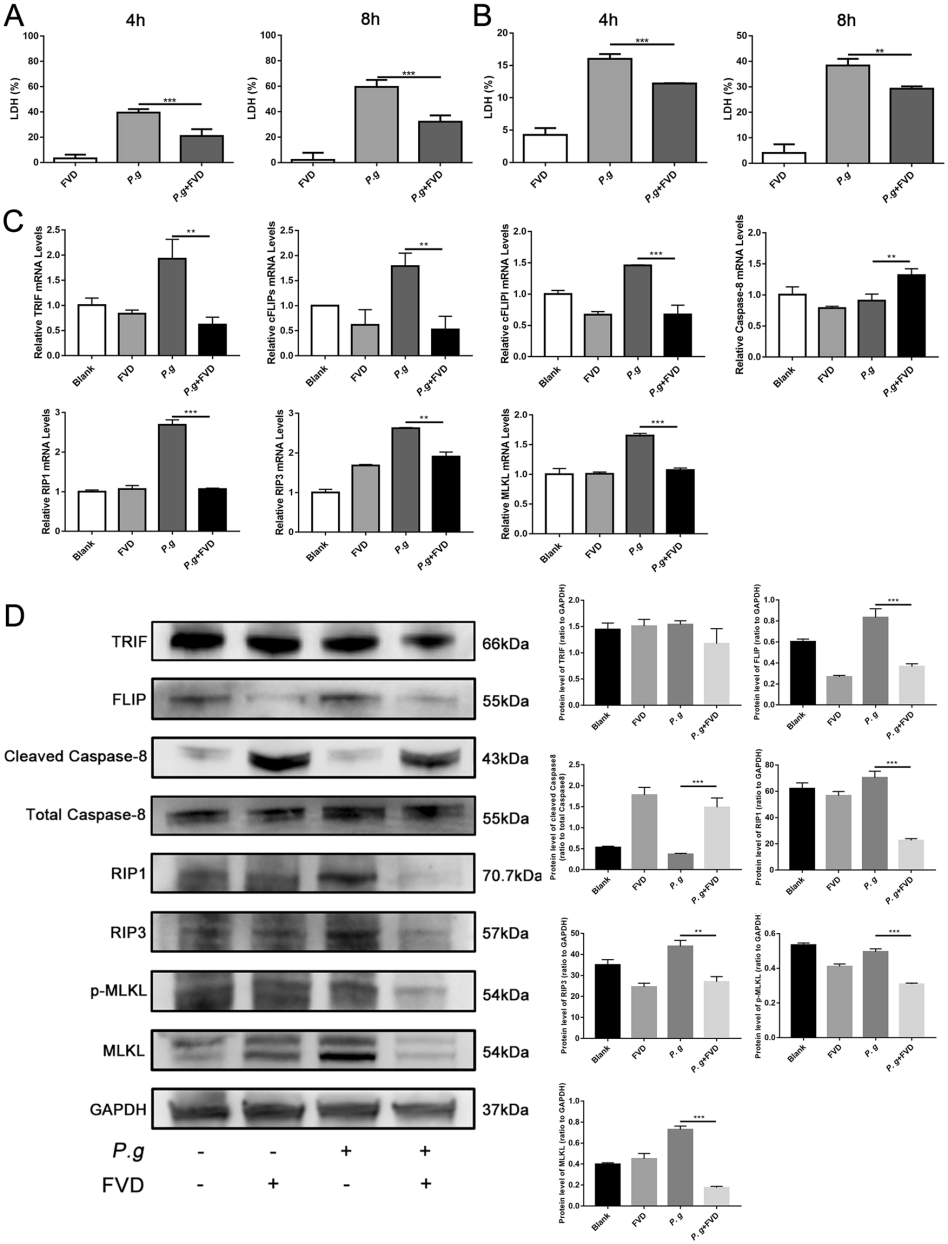
CDK9 inhibition reduced necroptotic cell death. (**A**) THP-1 cells were pretreated with FVD to inhibit CDK9 for 2 hours, and then stimulated with *P. gingivalis*. Expression of lactate dehydrogenase in the cell supernatants were assessed by LDH assay at 4- and 8-hours post-infection to represent cell death. (**B**) Same treatment was performed on PBMCs, and the expression of lactate dehydrogenase were detected. (**C**) The RNA levels of THP-1 cells related to necroptosis including TRIF, cFLIP_S_, cFLIP_L_, caspase-8, RIPK1, RIPK3 and MLKL were detected by real-time PCR. (**D**) The translational level was further detected by western blot. The images were collected from different gels with the same amount of samples. (**p < 0.01; ***p < 0.001).

CDK9 inhibition by FVD significantly reduced the level of lactate dehydrogenase (LDH), which indicates the onset of cell death, in both THP-1 monocytes (Fig. 5A) and PBMCs (Fig. 5B) at 4 and 8 hours after bacterial infection, indicating that CDK9 activation may trigger cell death in monocytes. Activation of MLKL resulted in its recruitment and oligomerization at phosphatidylinositol phosphates, leading to cell membrane rupture and cell death^29^. Blocking CDK9 by FVD significantly reduced MLKL transcription and protein expression; moreover, phosphorylated MLKL was significantly depressed after FVD pretreatment in THP-1 monocytes (Fig. 5C,D). In addition, FVD significantly decreased the level of RIPK3 after *P. gingivalis* stimulation, while its RHIM domain and kinase activity are needed to induce necroptosis^30,31^. Furthermore, FVD inhibited *P. gingivalis* up-regulated the level of RIPK1, a key adaptor in the cascade of death receptor^29^. TIR-domain-containing adapter-inducing interferon-β (TRIF), one crucial adaptor in the TLR3/4 and DAI-activated alternate necroptosis pathway, was also reduced. Therefore, our present results indicated that CDK9 participated in both the classical RIPK1-RIPK3-MLKL-mediated and the alternate TRIF-RIPK3- MLKL-mediated necroptosis.

**Figure 6:**
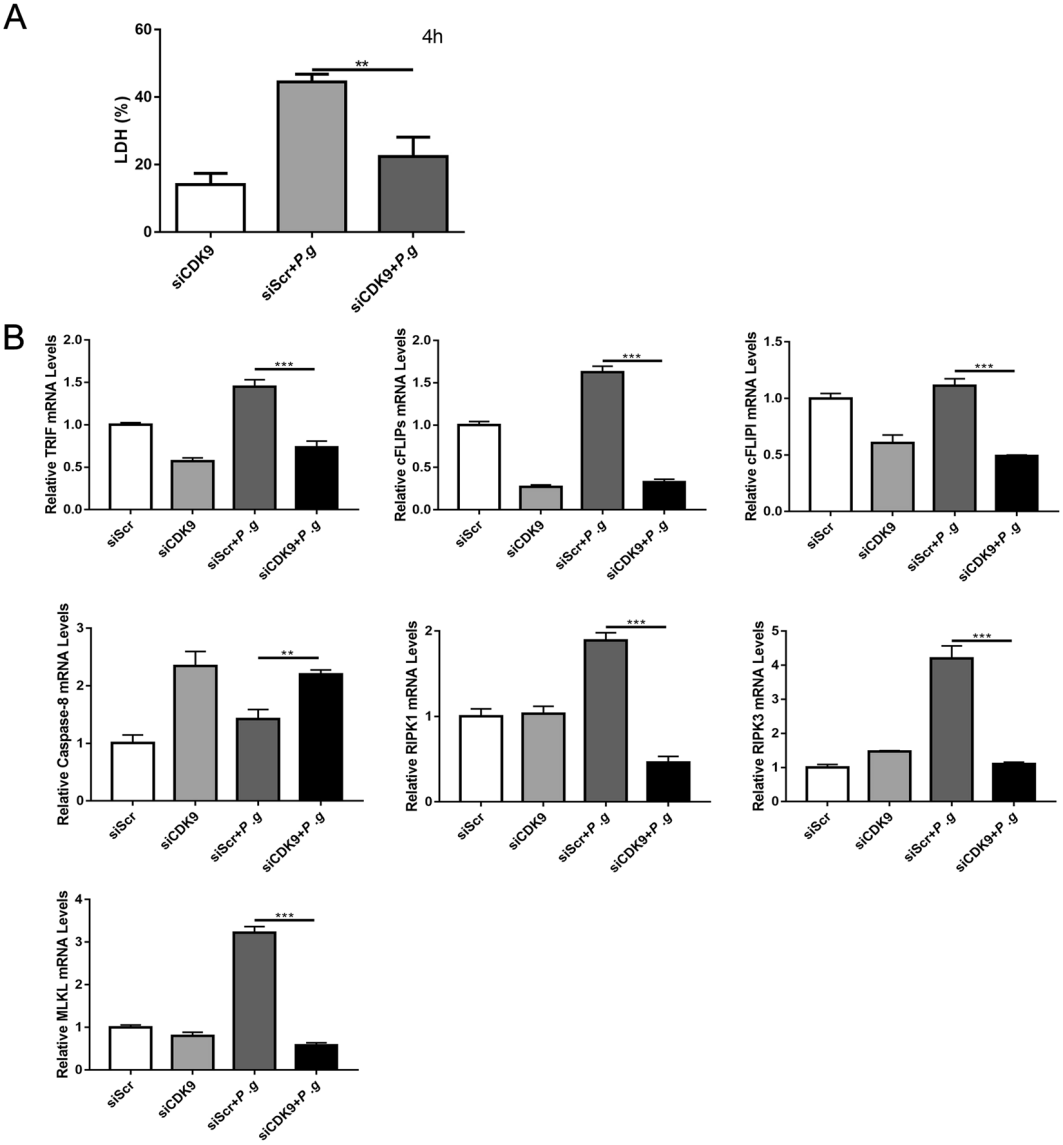
CDK9 knock-down altered genes in the network of cell death. THP-1 cells were transfected with lentivirus carrying CDK9 shRNA for 96 hours to inhibit CDK9, then stimulated with *P. gingivalis*. (**A**) The level of lactate dehydrogenase at 4 hours post-infection were detected by LDH assay. (**B**) The expression of molecules related to necroptosis were measured by qPCR. (**p < 0.01; ***p < 0.001).

As apoptosis and necroptosis are two inter-correlated death pathways in the immune response to microbial stimuli, caspase-8 and cFLIP regulate the fate of cells, i.e. cell survival or death; they also determine whether the immune cells undergo extrinsic apoptosis or necroptosis^32,33^. We next explored changes in caspase-8 and cFLIP after CDK9 inhibition. CDK9 inhibition by FVD in THP-1 monocytes upregulated caspase-8, while downregulated cFLIP after *P. gingivalis* incubation (Fig. 5C,D). The transfection of shRNA for THP-1 monocytes was performed as another way to inhibit CDK9, and the results of LDH levels and the expression of genes related to necroptosis were in consistence with the results of inhibition FVD pretreatment (Fig. 6). As difference in primary monocytes and monocytic cell lines may affect the onset of cell death, we conducted further experiments using PBMCs. Similarly, CDK9 inhibition modulated transcription of TRIF, cFLIP_S_, cFLIP_L_, caspase-8, RIPK1, RIPK3 and MLKL (See Supplementary Fig. S2). Such results indicated that CDK9 activation modulates immune responses to *P. gingivalis* by regulation of the cell death network.

### Blockade of CDK9 reduced the inflammation in the subcutaneous chamber model *in vivo*

**Figure 7:**
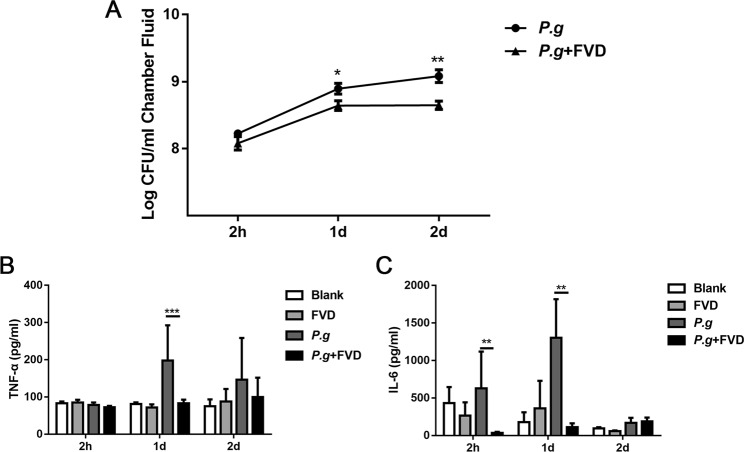
Inhibition of CDK9 influenced acute bacterial inflammation *in vivo*. The subcutaneous chambers were utilized to mimic *P. gingivalis* infection *in vivo*. FVD was injected intraperitoneally to block CDK9 2 hours before *P. gingivalis* (2 × 10^8^) inoculation in the chambers. **(A**) Numbers of viable *P. gingivalis* were enumerated on blood agar plates. (**B**,**C**) Level of TNF-α and IL-6 in the chamber fluid at 2, 24 and 48 h after infection were measured by ELISA. (*p < 0.05; **p < 0.01; ***p < 0.001).

We next investigated the biological significance of the FVD-mediated inhibition of CDK9 on *P. gingivalis*-induced immune responses *in vivo*. FVD pretreatment did not affect bacteria counts 2 hours after injection. Viable *P. gingivalis* cells showed a surge of growth at 24 h after injection, while less growth of bacteria was observed in the FVD group. Bacteria counts further climbed 48 h after bacterial infection in the *P. gingivalis* group, whereas bacterial numbers in the FVD group did not alter 48 h post-injection (Fig. 7A).

Elevation of TNF-α in diseased periodontal tissues is related to proliferation of anaerobic pathogens, leading to persistence of *P. ginigvalis* and prolongation of inflammation^34,35^. No difference was observed in TNF-α production as early as 2 h, and ~ one-fold increase in TNF-α can be observed 24 h post-infection, while FVD pretreatment dampened such increase in TNF-α production (Fig. 7B). Similarly, FVD pretreatment significantly reduced IL-6 production in the subcutaneous chamber fluids (Fig. 7C). Therefore, FVD treatment can inhibit the proinflammatory responses to *P. gingivalis in vivo*.

### Inhibition of CDK9 lessened the loss of periodontal tissues

**Figure 8:**
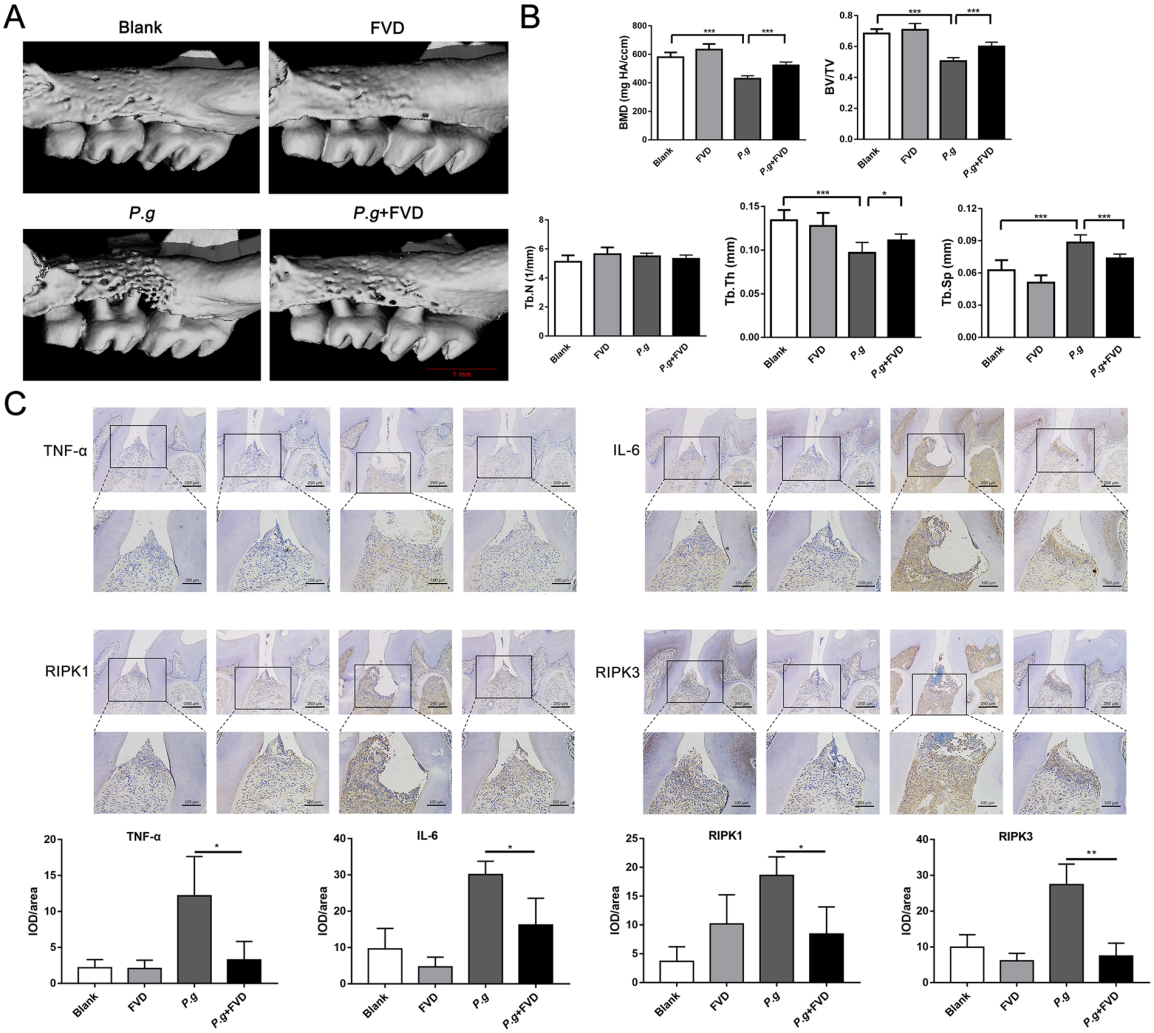
Inhibition of CDK9 alleviated the loss of periodontal tissues. Chronic periodontitis was established by silk ligation and *P. gingivalis* inoculation. FVD was injected intraperitoneally to block CDK9 2 h before *P. gingivalis* inoculation. (**A**) The level of alveolar bone absorption was measured by μCT from cementoenamel junction to alveolar bone crest. (**B**) Bone resorption was evaluated by several indexes of bone density. (**C**) Expression of TNF-α, IL-6, RIP1 and RIP3 in periodontal tissues were assessed by immunohistochemistry. (*p < 0.05; ***p < 0.001).

Chronic periodontitis is the inflammatory response to anaerobes in the periodontal tissue, which leads to the breakdown of the periodontal tissue and loss of resident periodontal ligament fibroblasts^36^. To explore the role of CDK9 in the development of periodontitis, silk ligation and *P. gingivalis* infection was utilized to mimic chronic periodontitis as described by Ke, *et al*.^26^, and FVD or DMSO was injected intraperitoneally 2 h before bacterial injection. From the results of micro-CT, FVD treatment reduced the loss of periodontal tissues. *P. gingivalis* inoculation resulted in resorption of alveolar bone, and FVD treatment mitigated the bone loss (Fig. 8A). Decrease in the bone mineral density (BMD), bone volume/total volume (BV/TV) and trabecular thickness (Tb.Th) of the alveolar bone was found in correlative analysis of bone density, whereas increase in trabecular separation (Tb.Sp) was observed after bacteria inoculation; therefore, FVD treatment mitigated bone resorption after bacterial infection (Fig. 8B).

We further assessed effects of FVD on inflammation and RIPK3-MLKL-mediated necroptosis in periodontal tissues. *P. gingivalis* inoculation leaded to accumulation of IL-6- and TNF-α-positive cells in periodontal tissue, while FVD treatment markedly reduced inflammatory cells in the periodontal tissue. Similarly, less RIPK1 and RIPK3 positive cells were found in FVD pretreatment group (Fig. 8C,D). Such results implied that CDK9 activation in periodontal tissue leaded to increased inflammation and necroptotic cell death and its inhibition may delay the occurrence and development of periodontitis.

## Discussion

CDK9, one important transcriptional regulator, can modulate NF-kB activity in response to proinflammatory stimuli^19,37,38^. In addition, CDK9 activation has also been implicated in osteoclast differentiation and bone resorption function^39^. To gain insights into the pathologic role of CDK9 in the periodontitis, we first investigated the expression of CDK9, Brd4 and RNA Polymerase II, which coordinate in modulating gene transcription. Furthermore, we explored the role of CDK9 in the periodontitis progress by inhibition with FVD or knock-down with shRNA. Our present study not only confirmed that CDK9 participated in the innate immune response, but also discovered a new mechanism through regulating the RIPK3-MLKL-mediated necroptosis in inflammatory diseases.

Blockage or knock-down of CDK9 in monocytes reduced inflammatory responses to anaerobic *P. gingivalis*, a result of dampened transcription in the gene promoters, similar dampened innate immune responses in the bacterial infection has been reported in Brd4 blockage by BET inhibitor I-BET^40,41^ or conditional knock-out of Brd4 in myeloid-lineage cells^42^, and topoisomerase 1 inhibition^16^. Despite anaerobic Gram-negative bacteria, *P. gingivalis* show pathogenic factors distinct from other Gram-negative bacteria. Generally, Gram-negative bacteria are recognized by TLR4, while *P. gingivalis* can be recognized by TLR2, TLR4, or both^43^, and TLR2 contributes to the periodontal bone absorption following *P. gingivalis* infection *in vivo*^44^. In addition, TNF-knockout mice are less susceptible to periodontal bone loss in animals with oral infection of *P. gingivalis*^43^. TLR signaling pathway activation triggered a cascade of adaptor molecules to initiate the pro-inflammatory gene transcription upon microbial stimuli. Regulation of Pol-II-mediated pausing is one principal target during transcriptional control^45^, and a vast array of transcription factors, including NF-κB^46^, c-Myc^47^, as well as hypoxia inducible factor-1α have been implicated in such process^48^. The extensive release of TLR-triggered CDK9-modulated inflammatory mediators may damage the host by exacerbating inflammation and crippling periodontal supporting tissues. Therefore, the reduced cytokine in the two *in vivo* experiments may account for the reduced alveolar bone loss after CDK9 inhibition. As multiple bacteria are involved in the process of periodontitis development and several pathogens used different strategies to invade periodontal tissues, experiments using other peri-pathogenic bacteria can further our knowledge of the pathological role of CDK9 in the development of periodontitis.

Owing to the importance of CDK9 in the embryogenesis, CDK9 knockout or its binding partner cyclin T2, are embryonically fatal to mouse^49,50^. Future study using conditional genetic knockout mouse may provide more information about CDK9-mediated transcription modulation in the infection.

In nature, inflammatory response may help defense cells to recruit more defense cells into the infection loci and to sustain the infection; however, in confront of over-growth of pathogens, the host defense cells in susceptible subjects may undergo several programmed cell deaths, e.g. RIPK3-MLKL-mediated necroptosis^51^, caspase-1 dependent pyroptosis after its activation by inflammasomes^52^ and apoptosis^53^. Once the extracellular and intracellular danger signals were sensed by various PRRs, i.e. TLRs on the cell membrane^17^ and NOD like receptors (NLRs) in the cytosol^54^, a cascade of adaptor molecules may trigger a diverse inflammatory response in the nuclei, which is characterized by a concerted activation of gene transcription. In the process of transcription pausing, CDK9 interacts with Brd4 to phosphorylate the RNA Pol II C-terminal domain^55^; in addition, it also phosphorylates the DRB sensitivity-inducing factor (DSIF) and negative elongation factor (NELF)^56^, leading to its dissociation from the RNA polymerase. Therefore, the couple of Brd4 and CDK9 may participate in the transcription of a variety of molecules, which are closely related to programmed cell death like apoptotic, necroptotic and pyroptotic cell death. Indeed, the gene microarray data revealed that CDK9 knock-down affected cell death including necroptosis, apoptosis and ferroptosis, which is featured by excessive pileup of reactive oxygen species and generation of lipid peroxidation products and can be blocked by iron chelator deferoxamine^57^.

Inhibition of CDK9 contributes to antitumoral effects by suppressing the pro-survival inflammatory genes and enhancing pro-apoptotic gene transcription including caspase-8 and -9, while reducing anti-apoptotic genes, such as Mcl-1, XIAP, BCL6 and BTG1^47,58^. In addition, CDK9 also contributed to altered apoptosis in non-tumor cells. For example, treatment of neutrophils with flavopiridol contributes to increased apoptosis with downregulation of the anti-apoptotic genes Mcl-1 and unaffected expression of Bcl2A^59^. Our present study confirmed that CDK9 knock-down upregulated caspase-8 level after *P. gingivalis* treatment, indicating flavopiridol pretreatment shifted the balance of survival and cell death to favor apoptosis.

Necroptosis play a vital role in maintaining tissue homeostasis and orchestrating bacterial invasion^60^. Three necroptotic death pathways have been discovered, including the RHIM-containing RIPK1, TRIF and Z-DNA binding protein 1-mediated pathway^61^. In our present study, CDK9 inhibition or knock-down significantly reduced RIPK1, RIPK3 and TRIF levels after bacterial stimulation, thereby may reduce necroptosis during bacterial infection. Indeed, reduced pMLKL, the definite marker of necroptosis, was observed in *P. gingivalis* treated monocytes after CDK9 inhibition or knock-down. In addition, decreased RIPK1 and RIPK3 can be observed in the periodontal tissue in the experimental periodontitis model after FVD treatment, further indicating that CDK9 may modulate necroptosis during periodontitis progression. However, biological activity of RIPK1 depends on the context of stimuli. RIPK1 is involved in three distinct complexes following ligation of TNF-α with its receptor^62^. Complex I promotes activation of NF-κB pathway to facilitate inflammation as well as survival; Complex II activates the noninflammatory apoptosis; Complex III formation leads to pro-inflammatory necroptosis^61^. Previously, there are no reports regarding the role of CDK9 in regulating RIPK1, TRIF, RIPK3 and MLKL. Our study extends the importance of CDK9 and Brd4 coordination in regulating necroptosis by both the classical RIPK1-mediated and alternate TRIF-mediated necroptosis.

Life and death of cells are closely entangled events during infection. Several key molecules finely tune the fate of cells in response to various stimuli. The presence of the cFLIP in the FAS-associated death domain (FADD)-caspase-8-cFLIP complex decides the fate of cells in response to stimuli^32^. Caspase-8 shows two opposing roles–promoting cell death by the extrinsic apoptosis pathway and preventing RIPK3-mediated necroptosis while not inducing apoptosis. It functions proteolytically with FLICE-like inhibitory protein long (FLIP_L_)^63^. cFLIP has two isoforms due to the post-transcriptional mRNA splicing. The cFLIP_s_ protein can dampen caspase-8 activity and block procaspase-8 activation, while cFLIP_L_ may regulate the extent of activation of procaspase-8 and its substrate specificity^64^. Moreover, selective CDK9 inhibition suppressed activity of cFLIP and Mcl-1 to enhance TRAIL-induced apoptosis^65^. Our present study further demonstrated that by down-regulation of both cFLIP_s_ and cFLIP_L_ and upregulation of caspase-8, thereby CDK9 inhibition attenuated *P. gingivalis*-induced necroptosis.

Monocytic cell lines, including THP-1 and U937 has been widely applied to mimic monocytes in cell culture models in exploring mechanism of inflammation^66^ and cell death, such as apoptosis^67^, pyroptosis^68^ and necroptosis^69^. However, difference in primary monocytes and monocytic cell lines may influence the onset of cell death. Although we used PBMCs to confirm that CDK9 regulated necroptosis development during *P. gingivalis* invasion, further studies are still needed to further confirm its mechanism in regulating necroptosis.

Homeostasis in the periodontium is maintained by a dynamic balance between innate immune system and biofilms around the periodontal tissue. Our previous studies indicated that RIPK3-MLKL-mediated necroptosis participated in the progress of periodontitis^26,70^. With its profuse release of DAMPs into the periodontal tissue, necroptosis may exacerbate the periodontal inflammation. In addition, microRNA-214 modulates necroptosis via ATF4 in diabetes-associated periodontitis^71^. Our present study further indicated that RIPK3-MLKL-mediated necroptosis in periodontal tissues can be regulated by CDK9-mediated transcription modulation.

In conclusion, CDK9 plays a critical role in the bacteria-induced inflammatory disease development. We characterized a novel mechanism involving RIPK3-MLKL-mediated necroptosis. CDK9-mediated transcription regulates the network of cell survival, apoptosis and necroptosis by targeting key molecules involving in such process, like RIPK1, caspase-8 and cFLIP. By modulating both the TRIF-dependent alternate and RIPK1-dependent classic necroptosis, CDK9 inhibition may enhance survival of immune cells and reduce release of DAMPs during periodontitis progress. Although inhibiting inflammatory response may help reduce periodontal bone resorption and inflammation caused by *P. gingivalis* in the present study, clinical application of CDK9 inhibitors should be cautious.

## Methods

### Samples

Gingival tissues were collected from 10 periodontitis adults and 10 heathy adults without periodontal diseases, the information of these patients was shown in Table 1. The gingiva of periodontitis adults was obtained from hopeless teeth with III° mobility that need to be extracted. All of the teeth had positive bleeding on probing with probing depth ≥6 mm or clinical attachment loss ≥6 mm. Gingiva of healthy adults was obtained from embedded and impacted wisdom teeth without inflammation during extraction. Gingiva samples from each individual (not mixed) were utilized for mRNA and protein analysis. Peripheral blood mononuclear cells (PBMCs) were separated from whole blood. The blood was collected from 3 adult volunteers without periodontitis. ~100 mL blood was got from each adult by BD vacutainer tubes with ethylenediaminetetraacetic acid through venipuncture.

**Table 1:** The demography and periodontal data of patients who donated the clinical samples.

Gender	Normal	Periodontitis
Sex (male/female)	6/4	4/6
Age	48.9 ± 6.72	48.1 ± 7.77
Average of Pocket Depth (mm)	1.5 ± 0.53	8.1 ± 2.60
Clinical Attachment Loss(mm)	0	7.3 ± 2.75
Gingival Index	0	2.075 ± 0.24

### Bacterial strains

*P. gingivalis* (ATCC 33277) was cultured in brain heart infusion (BHI) medium with yeast extract (50 mg/L), vitamin K3 (1 mg/L) and hemin (1 mg/L) at 37 °C in a humidified incubator anaerobically. The incubation of *P. gingivalis* detected by the optical density at 600 nm, and 1 was corresponding to 10^9^ CFU/ml.

### Cell culture

Human acute monocytic leukemia cell line (THP-1, ATCC) were cultured in RPMI1640 medium (Gibco, US) with 10% fetal bovine serum (FBS, Gibco), 1% penicillin/streptomycin solution (Hyclone, US) and 0.1% β-mercaptoethanol (Sigma, US) at 37 °C in a 5% CO_2_ humidified incubator. PBMCs were isolated using Ficoll as described by Corkum C P *et al*.^72^ and were cultured in RPMI1640 medium supplemented with 10% FBS and 1% penicillin/streptomycin.

### Cell infection model

THP-1 cells were seeded into 24-well plate with different treatment. Blank group was treated with PBS after pretreated with DMSO for 2 hours. CPT, TPT, JQ1 and FVD group were pretreated with CPT (5 nM, 50 nM, 500 nM), TPT (1 nM, 10 nM, 100 nM), JQ1 (50 nM, 250 nM, 500 nM) and FVD (4 nM, 40 nM, 400 nM) for 2 hours, respectively, and then with PBS. *P. gingivalis* group were treated with *P. gingivalis* (MOI = 40) after the pretreatment of DMSO for 2 hours. *P. gingivalis* and CPT, *P. gingivalis* and TPT, *P. gingivalis* and JQ1, *P. gingivalis* and FVD group received pretreatment of CPT, TPT, JQ1 and FVD for 2 hours respectively, and then were treated with *P. gingivalis*. Antibiotics were removed in all groups during treatment. The supernatant at 4 and 24 hours after the treatment of PBS or *P. gingivalis* was collected. Following the instructions, IL-6 and TNF-α in cells supernatants were measured by ELISA assay (Neobioscience, China).

THP-1 cells and PBMCs were also seeded into 24-well plate and divided into four groups. Blank group were pretreated with DMSO for 2 hours and then with PBS. FVD group were treated with PBS with the pretreatment with FVD (40 nM) for 2 hours. *P. gingivalis* group were treated with *P. gingivalis* (MOI = 40) with the pretreatment of DMSO for 2 hours. *P. gingivalis* and FVD group were treated with *P. gingivalis* after the pretreatment of FVD for 2 hours. The supernatant was collected at 4 and 24 hours for cytokine detection.

### Cell death model

THP-1 cells and PBMCs were seeded into 6-well plate and divided into four groups. Blank group were pretreated with DMSO for 2 hours and then with PBS. FVD group were treated with PBS with the pretreatment with FVD (40 nM) for 2 hours. *P. gingivalis* group were treated with *P. gingivalis* (MOI = 200) with the pretreatment of DMSO for 2 hours. *P. gingivalis* and FVD group were treated with *P. gingivalis* after the pretreatment of FVD for 2 hours. RNA was extracted 2 hours later, and protein extraction was performed 6 hours later.

### shRNA knockdown

THP-1 cells were cultured in 24-well plates. Lentiviral vectors carrying CDK9 shRNA or not were transfected into cells, with the assistance of Envirus (Engreen Biosystem). The expression of green fluorescence was observed at 96 h post-transfection. RNA and proteins were extracted to examine the expression of CDK9. THP-1 cells were seeded into 96-well plate and 6-well plate for cytotoxicity analysis and real-time PCR analysis, respectively. Cells were divided into four groups. NC group was transfected with non-targeting scramble and stimulated with PBS. NC and *P. gingivalis* group was transfected with non-targeting scramble and stimulated with *P. gingivalis*. CDK9 shRNA group was transfected with CDK9 shRNA and stimulated with PBS. CDK9 shRNA and *P. gingivalis* group was transfected with CDK9 shRNA and stimulated with *P. gingivalis*. After transfection for 96 hours, cells were stimulated with *P. gingivalis* and PBS, then 4 hours later, cytotoxicity analysis was detected by LDH assay. RNA was extracted at 2 hours post-stimulation.

### Mice

Female C57BL/6 mice were raised under a 12/12 h day/night cycle until 8 weeks with sterile food and water. All mice were adaptively fed for one week in the new environment before experiment.

### Murine experimental periodontitis model

The groupings included blank group, FVD group, *P. gingivalis* group, *P. gingivalis* and FVD group, and each group consisted of 15 mice. Blank group mice were treated with silk ligation and PBS injection. FVD group mice were treated with silk ligation and FVD injection. *P. gingivalis* group mice received experimental periodontitis modeling and PBS injection. *P. gingivalis* and FVD group received experimental periodontitis modeling and FVD injection. Experimental periodontitis model was established as described by Julie Marchesan *et al*.^73^. Briefly, silk (5-0) soaked in solution with *P. gingivalis* for one night was placed around the maxillary second molars to contribute a carrier for *P. gingivalis*. After that, 10 μl of *P. gingivalis* (2 × 10^8^) were injected into six sites of the maxillary second molars, including mesial buccal, buccal, distal buccal, mesial palatine, palatine and distal palatine sides every two days. Intraperitoneal injection of FVD (5 mg/kg) or PBS was performed 2 hours before the bacterial injection. After 10 days, all mice were executed, and the maxillary bones with three molars were harvested for further analysis.

### Murine acute inflammation model

180 mice were divided into four groups as described before in murine periodontitis experiment. Each group consisted of 45 mice, with 15 mice treated for 2 h, 15 mice treated for 24 h and 15 mice treated for 48 h. Middorsal subcutaneous implantation of coil with 5 mm diameter was performed as described by Genco et. al. to form a chamber^74^. After 10 days, when the wound was healed, PBS were injected into chambers with PBS pretreatment 2 hours before by intraperitoneal injection in blank group. Mice in FVD group were injected with PBS and the pretreatment of FVD (5 mg/kg) by intraperitoneal injection. While in *P. gingivalis* group, *P. gingivalis* (2 × 10^8^ CFU in 100 μl of PBS) were injected into the chambers to cause acute inflammation and PBS were pre-injected. The group of *P. gingivalis* and FVD were treated with *P. gingivalis* solution and the pretreatment of FVD. The chamber exudates were collected at 2, 24 and 48 h after PBS or FVD infection, and were used for the evaluation of bacterial counts and examination of IL-6 and TNF-α by ELISA assay.

### Micro-CT assessment

The maxillary bones with molars of mice for experimental periodontitis experiment were fixed in 4% paraformaldehyde overnight, and then were examined by a vivaCT micro-computed tomography scanner (Scanco Medical, Bassersdorf, Switzerland) at 10.5 μm resolution. The 3Dvolumes were reconstructed. Volumetric and morphometric analyses including BMD, BV/TV, Tb.N, Tb.Th and Tb.Sp were performed as described^75^.

### Immunohistochemical analysis

Samples including mice maxillary bones and human gingiva tissues were fixed in 4% paraformaldehyde overnight. After the decalcifications of bone tissue, all samples were conducted by dehydrating and embedding. The immunohistochemistry was performed as described by Ke X^26^. Briefly, the thickness of tissue slides was 4μm. After dewaxing and antigen retrieval, slides were blocked in PBST with 3% BSA. Incubation with the primary antibodies was carried out at 4 °C overnight by rabbit anti-TOP1, rabbit anti-BET, rabbit anti-CDK9, rabbit anti-RIPK1/RIP, rabbit anti-RIP3, rabbit anti-IL-6 (Abcam, USA) and rabbit anti-TNF-α (Abcam, USA). After incubation with streptavidin-HRP-conjugated secondary antibody for 30 min, chromogenic reagent kit was used. Slides were then dehydrated, hematoxylin immersed, differentiated and mounted with neutral gum.

### Cytotoxicity assay

THP-1 cells and PBMCs were seeded into 96-well plate. Then cells were divided into four groups, and treated as cell death model. After treatment with bacterial infection or PBS for 4 and 24 hours, the LDH levels were assessed by LDH assay (Promega, USA). The optical absorbance at 490 nm was detected. The absorbance value of RPMI 1640 medium served as benchmark, and the absorbance value of completely lysed cells was regarded as the maximal LDH release. The practical value detected by LDH assay was corrected to indicate cytotoxicity.

### RNA extraction and quantitative real-time PCR analysis

RNA extraction and analysis were performed as described^26^. Total RNA was extracted by RNA extraction kit (Tiangen, China) after treatment with PBS or *P. gingivalis* for 2 hours or from the samples of gingiva tissue which were collected clinically. RNA was quantified by Nanodrop Spectrophotometer and reverse-transcribed by Superscript II (Takara, Japan). Real-time PCR was performed using SYBR Green Master MIX (ABI, USA). The primers can be found as Supplementary Table S1.

### Microarray expression analysis

Total RNA from THP-1 cells infected with CDK9 shRNA or non-targeting scramble shRNA was stored at −80 °C. After determination the quantity, quality and integrity of RNA, each sample were linearly amplified and labeled with Cy3-UTP, and purified by RNeasy Mini Kit (Qiagen). Following determining concentration and specific activity of labeled cRNAs by NanoDrop ND-1000, cRNA fragmentation was finished by addition of 10 × blocking agent (11 μL) and 25 × fragmentation buffer (2.2 μL). The mixture was incubated at 60 °C for 30 min, followed by dilution with 2 × GE hybridization buffer (55 μL). After dispensing hybridization solution (100 μL) into the gasket slide, the solution was assembled into microarray slides. After incubation at 65 °C in the Agilent Hybridization Oven for 17 hours, the slides were rinsed, fixed and scanned with the Agilent DNA Microarray Scanner (part number G2505C). Original fluorescent data were read by Agilent Feature Extraction software, and GeneSpring GX v12.1 (Agilent Technologies) was utilized for quantile normalization and data processing. Fold change larger than 2 between groups were determined as differential expression. Kyoto encyclopedia of genes and genomes (KEGG) pathway database was utilized to unveil the mechanism of CDK9-mediated transcription modulation during bacterial infection.

### Protein extraction and western blot analysis

The extraction of protein was performed as described^76^. Proteins were separated by 12% Bis-Tris Plus gels (Genscript, China), transferred to PVDF membrane (Millipore, USA) by 350 mA for 90 min, and blocked for 1 hour at room temperature. Primary antibodies used to probe blots were mouse anti-TICAM-1 (sc-514384, mAb, Santa, USA), mouse anti-FLIPS/L (sc-5276, mAb, Santa, USA), rabbit anti-cleaved caspase-8 (9496, mAb, CST, USA), rabbit anti-total caspase-8 (ab108333, mAb, Abcam, USA), rabbit anti-RIPK1/RIP1 (NBP1-77077, polyclonal Ab, Novus, USA), rabbit anti-RIP3 (ab152130, polyclonal Ab, Abcam, USA), rabbit anti-MLKL (ab184718, mAb, Abcam, USA), rabbit anti-p-MLKL (phospho S358) (ab187091, mAb, Abcam, USA), rabbit anti-TOP1 (20705-1-AP, polyclonal Ab, Proteintech, USA), rabbit anti-BET (13232, mAb, CST, USA), rabbit anti-CDK9 (2316, mAb, CST, USA) and rabbit anti-GAPDH (MB001, mAb, Bioworld, USA), which were incubated overnight at 4 °C. Subsequently, HRP-conjugated secondary antibodies including anti-rabbit and anti-mouse antibodies (Fcmacs, China) were incubated for 1 hour. Blots were then visualized by a chemiluminescent imaging system (Tanon, Shanghai, China).

### Ethics statement

The experimental protocols were approved by the Medical Ethics Committee of Nanjing Stomatological Hospital, Medical School of Nanjing University, and the ethics approval number was 2017NL-012(KS). The informed consent was obtained from all participants included in the study. All experiments involving human participants or mice were carried out in accordance with relevant guidelines and regulations.

### Statistical analysis

Data were evaluated by one-way ANOVA and t-test by SPSS 20.0, t-test was only for the comparation of two group. Data were expressed as means ± standard deviation (SD). P <0.05 was taken as the level of significance.

## Supplementary information

Supplementary information

## Data Availability

The data sets generated during and/or analyzed during the current study are available from the corresponding author upon reasonable request.
