# Supplementary material for: Transcription modulation by CDK9 regulates inflammatory genes and RIPK3-MLKL-mediated necroptosis in periodontitis progression
Source: Sci Rep. 2019 Nov 22;9:17369. doi: 10.1038/s41598-019-53910-y (PMC6874675; doi:10.1038/s41598-019-53910-y)
Supplement: Supplementary file 1 — Supplementary information [file 41598_2019_53910_MOESM1_ESM.pdf]

Transcription modulation by CDK9 regulates inflammatory genes and RIPK3-MLKL-mediated necroptosis in periodontitis progression

Jiao Li[1,2], Jiahong Shi[2,3], Yue Pan[2], Yunhe Zhao[2], Fuhua Yan[2,3], Houxuan Li[2,3,*], Lang Lei[1,2*]

1. Department of Orthodontics, Nanjing Stomatological Hospital, Medical School of Nanjing University, Nanjing, China.
2. Central Laboratory of Stomatology, Nanjing Stomatological Hospital, Medical School of Nanjing University, Nanjing, China.
3. Department of Periodontics, Nanjing Stomatological Hospital, Medical School of Nanjing University, Nanjing, China.

Materials

Cell culture

RAW264.7 macrophages were cultured in Dulbecco's minimal essential medium (DMEM, Gibco), with 10% FBS and 1% penicillin/streptomycin solution at 37°C in a 5% CO_2 humidified incubator.

Cell infection model

RAW264.7 cells were seeded into 24-well plate with different treatment. Blank group was treated with PBS after pretreated with DMSO for 2 hours. CPT, TPT, JQ1 and FVD group were pretreated with CPT (5 nM, 50 nM, 500 nM), TPT (1 nM, 10 nM, 100 nM), JQ1 (50 nM, 250 nM, 500 nM) and FVD (4 nM, 40 nM, 400 nM) for 2 hours, respectively, and then with PBS. LPS group were treated with *E. coli* LPS (1 μg/mL) after the pretreatment of DMSO for 2 hours. LPS and CPT, LPS and TPT, LPS and JQ1, LPS and FVD group received pretreatment of CPT, TPT, JQ1 and FVD for 2 hours, respectively, and then were treated with LPS, the antibiotics were removed in all groups no matter with LPS or not. The supernatant at 4 and 24 hours after the treatment of PBS or LPS was collected. IL-6 and TNF-α in cells supernatants were detected by ELISA assay (Neobioscience, China) according to manufacturer's instructions.

Supplementary Figure S1

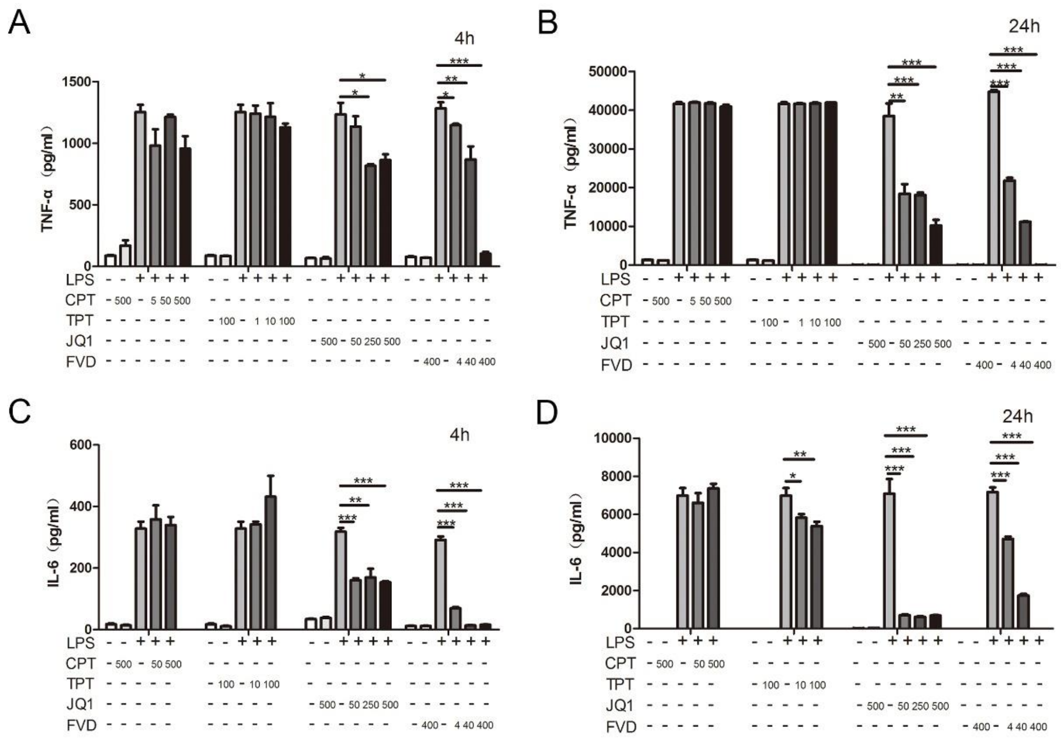

Supplementary Figure S2

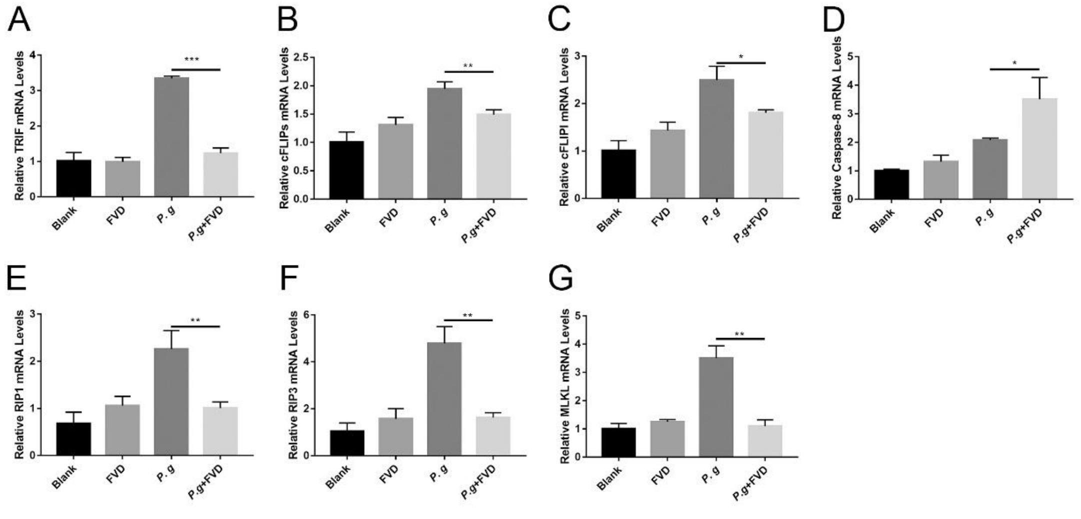

Figure Legends

Supplementary Figure S1. **Transcription modulation affected expression of inflammatory cytokines.** RAW264.7 cells were pretreated with CPT, TPT, JQ1 and FVD for 2 hours and stimulated with *E. coli* lipopolysaccharide (1 μg/mL). Production of (A, B) TNF-α and (C, D) IL-6 in culture supernatants were detected by ELISA at 4- and 24-hours post-stimulation. (*, $p<0.05$; **, $p<0.01$; ***, $p<0.001$).

Supplementary Figure S2. **CDK9 inhibition reduced necroptosis induced by *P. gingivalis.*** PBMCs were pretreated with FVD for 2 hours, and then stimulated with *P. gingivalis*(MOI=200). Necroptosis-related genes including TRIF, $cFLIP_S$, $cFLIP_L$, caspase-8, RIPK1, RIPK3 and MLKL were detected by real-time PCR.

Supplementary Table S1. Primer sequences.

Primer name	Forward primer sequence (5’-3’)	Reverse primer sequence (5’-3’)
CDK9	GTTCGAGTACTTGGCACCAC	GTCTCCACGATGCAAGTCAC
Brd4	ACCTCCAACCCTAACAAGCC	TTTCCATAGTGTCTTGAGCACC
TOP1	AAGGTCCAGTATTTGCCCCAC	ATTCATGGTCGAGCATTTTTGC
TRIF	AGCGCCTTCGACATTCTAGGT	AGAACCATGGCATGCAGGA
cFLIPs	TTGGAAATTGTTCCATGTGATT	GCAACAAGAAAGGGCTAAACA
cFLIPl	GCTCACCATCCCTGTACCTG	CAGGAGTGGGCGTTTTCTT
Caspase-8	TTTCTGCCTACAGGGTCATGC	GCTGCTTCTCTCTTTGCTGAA
RIP1	GGCATTGAAGAAAAATTTAGGC	TCACAACTGCATTTTCGTTTG
RIP3	CTCTCTGCGAAAGGACCAAG	CATCGTAGCCCCACTTCCTA
MLKL	CTCTTTCCCCACCATTTGAA	TCATTCTCCAGCATGCTCAC
Actin	GTGGGGCGCCCCAGGCACCA	CGGTTGGCCTTGGGGTTCAGGGGG G

The original unprocessed images for blot membranes were supplemented. The target panel was marked in red.

Ⅰ. Original images for Figure 1B.

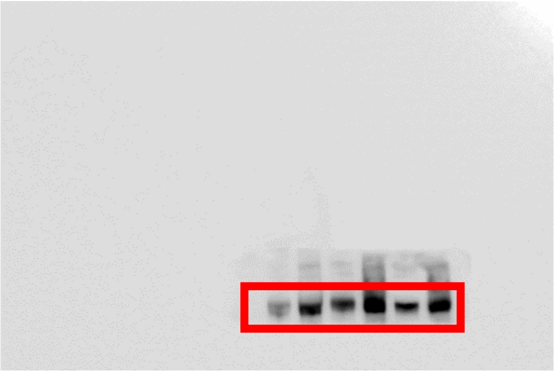

TOP1

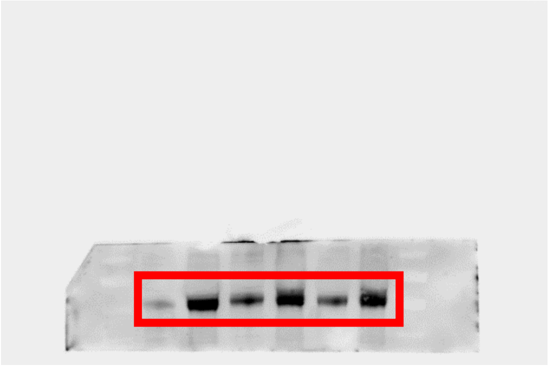

BET

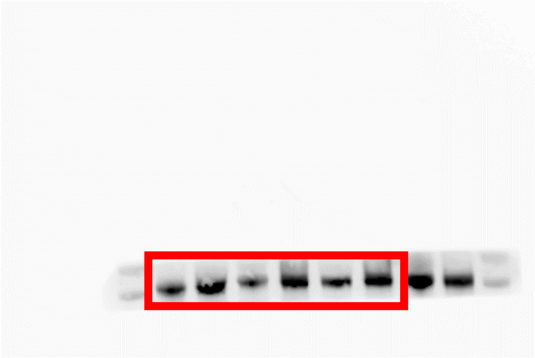

CDK9

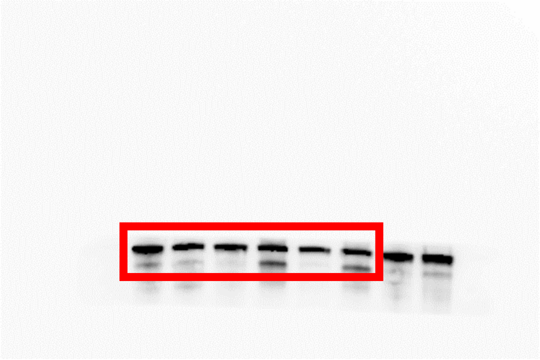

GAPDH

Ⅱ. The original images for Figure 4A.

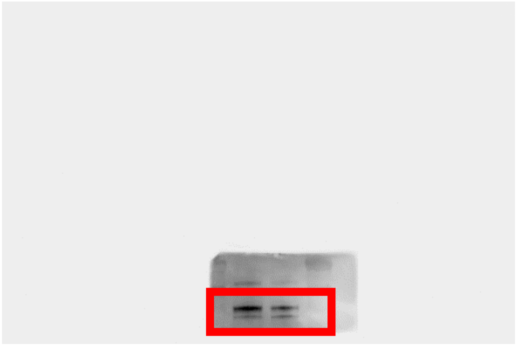

CDK9

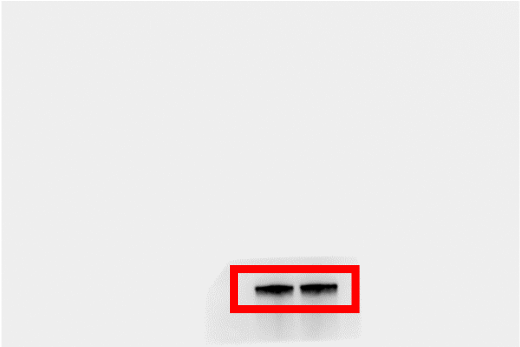

GAPDH

Ⅲ. The original images for Figure 5D.

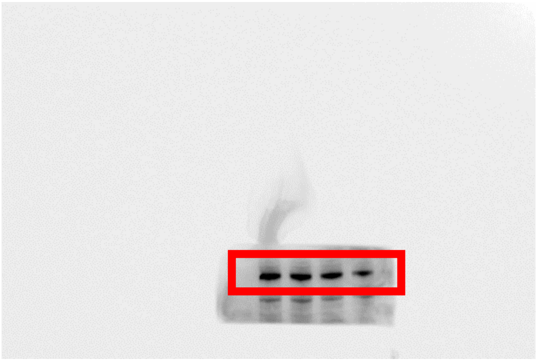

TRIF

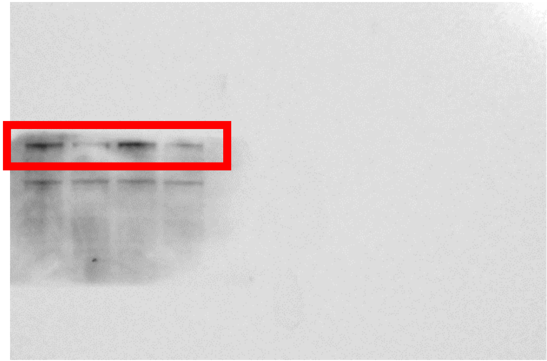

FLIP

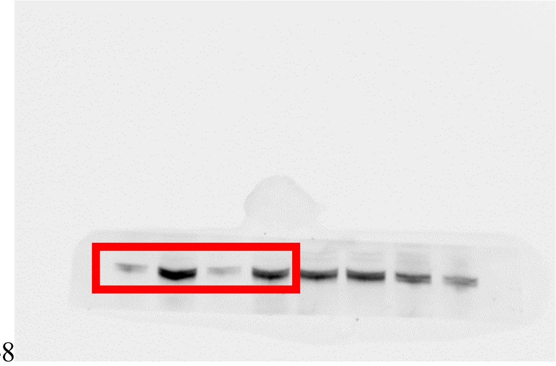

Cleaved Caspase-8

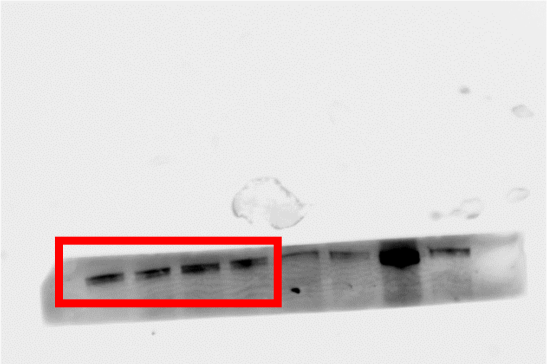

Total Caspase-8

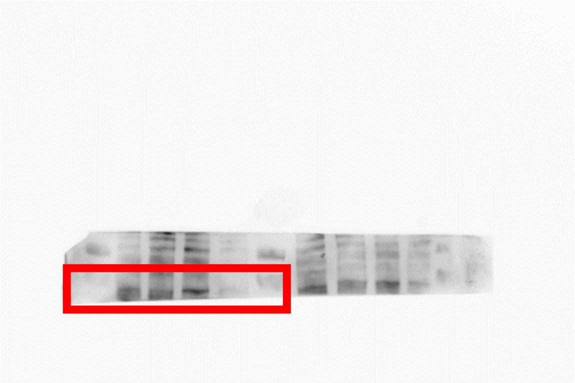

RIP1

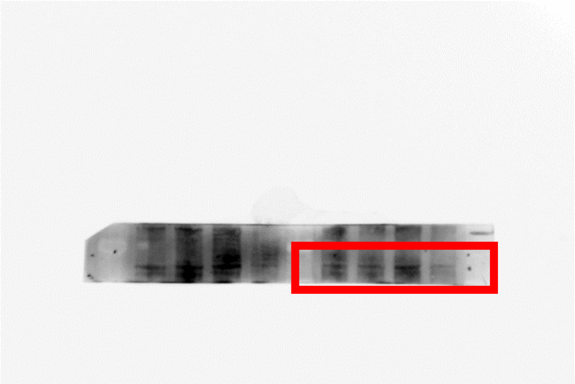

RIP3

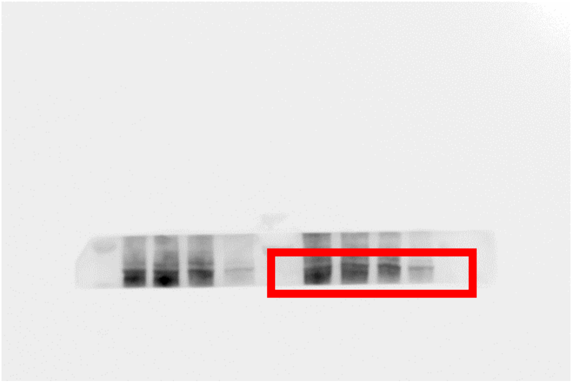

p-MLKL

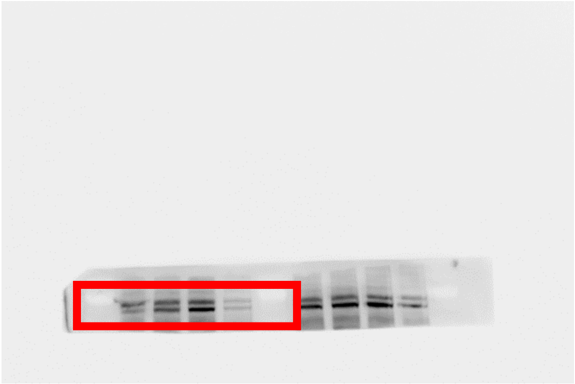

MLKL

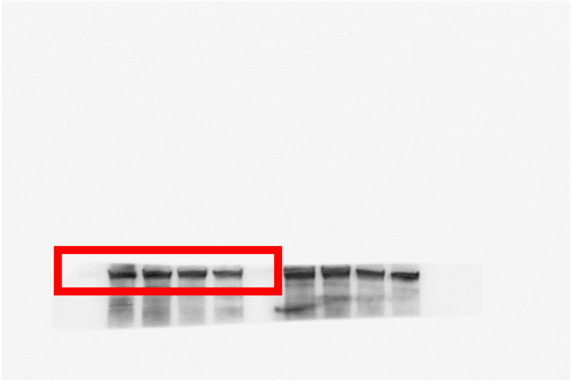

GAPDH
